# (Acetyl­acetonato-κ^2^ *O*,*O*′)(phthalo­cyaninato-κ^4^ *N*)(phen­an­throline-κ^2^ *N*,*N*′)erbium(III)

**DOI:** 10.1107/S1600536812003972

**Published:** 2012-02-04

**Authors:** Hong-Feng Li, Peng-Fei Yan

**Affiliations:** a School of Chemistry and Materials Science, Heilongjiang University, Harbin 150080, People’s Republic of China

## Abstract

The title complex, [Er(C_32_H_16_N_8_)(C_5_H_7_O_2_)(C_12_H_8_N_2_)], possesses a mirror plane and the asymmetric unit is half of the mol­ecule. The Er^III^ cation, lying on the mirror plane, is eight-coordinated by two O atoms from acetyl­acetone, two N (N_phen_) atoms from 1,10-phenanthroline and four isoindole N (N_iso_) atoms from the phthalocyanine ligand in an anti­prismatic geometry. The Er—N distances are in the range 2.376 (5)–2.529 (4) Å and the Er—O distance is 2.272 (3) Å. Notably, the Er—N_iso_ bonds are shorter than the Er—N_phen_ bonds, but longer than the Er—O bonds.

## Related literature

For background to phthalocyanines, see: Kuznetsova *et al.* (2002 ▶); Kalashnikova *et al.* (2007 ▶). For a similar erbium complex, see: Zugle *et al.* (2011 ▶).
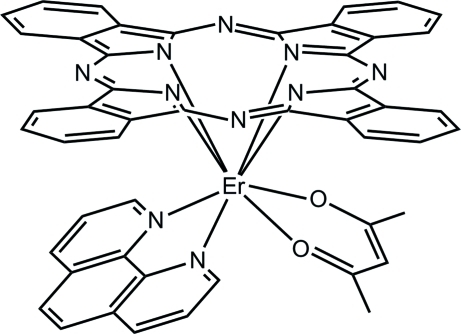


## Experimental

### 

#### Crystal data

[Er(C_32_H_16_N_8_)(C_5_H_7_O_2_)(C_12_H_8_N_2_)]
*M* *_r_* = 959.10
Monoclinic, 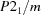

*a* = 9.913 (2) Å
*b* = 16.887 (3) Å
*c* = 12.622 (3) Å
β = 106.72 (3)°
*V* = 2023.7 (7) Å^3^
*Z* = 2
Mo *K*α radiation
μ = 2.13 mm^−1^
*T* = 293 K
0.28 × 0.22 × 0.17 mm

#### Data collection

Bruker SMART1000 CCD diffractometer
Absorption correction: multi-scan (*SADABS*; Sheldrick, 2003 ▶) *T*_min_ = 0.576, *T*_max_ = 0.696
19901 measured reflections
4769 independent reflections
3876 reflections with *I* > 2σ(*I*)
*R*_int_ = 0.050

#### Refinement

*R*[*F* ^2^ > 2σ(*F* ^2^)] = 0.033
*wR*(*F* ^2^) = 0.092
*S* = 1.13
4769 reflections
287 parameters
H-atom parameters constrained
Δρ_max_ = 2.01 e Å^−3^
Δρ_min_ = −2.24 e Å^−3^

### 

Data collection: *SMART* (Bruker, 2001 ▶); cell refinement: *SAINT-Plus* (Bruker, 2003 ▶); data reduction: *SAINT-Plus*; program(s) used to solve structure: *SHELXS97* (Sheldrick, 2008 ▶); program(s) used to refine structure: *SHELXL97* (Sheldrick, 2008 ▶); molecular graphics: *SHELXL97*; software used to prepare material for publication: *SHELXL97*.

## Supplementary Material

Crystal structure: contains datablock(s) global, I. DOI: 10.1107/S1600536812003972/vm2147sup1.cif

Structure factors: contains datablock(s) I. DOI: 10.1107/S1600536812003972/vm2147Isup2.hkl

Additional supplementary materials:  crystallographic information; 3D view; checkCIF report

## Figures and Tables

**Fig. 1.**
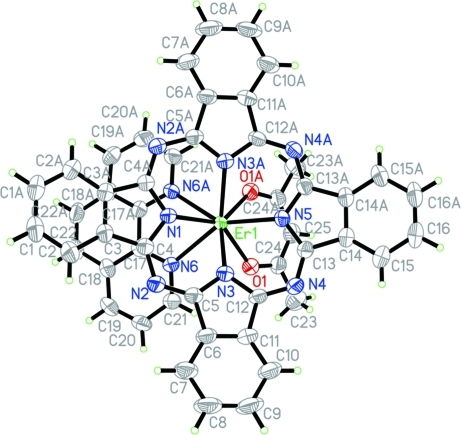
The molecular structure of the title compound, showing 50% probability displacement ellipsoids [symmetry code A: x, -y + 3/2, z].

**Fig. 2.**
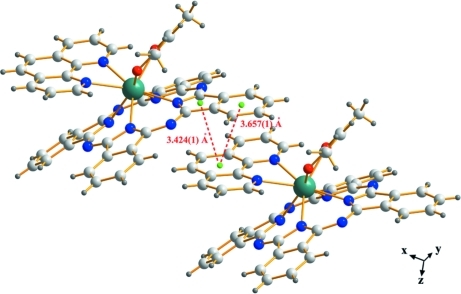
π–π interaction between the phthalocyanine isoindole group and the adjacent 1,10-phenanthroline ring as shown by the red dashed line [symmetry code: 1 + x, y, z].

## supplementary crystallographic information

### Comment

In recent years, lanthanide complexes with organic ligands have been widely used in areas such as fluorescent materials, electroluminescence and as fluorescence probes. Phthalocyanines (*Pc*) are of the most famous macrocyclic compounds that possess interesting physical and chemical properties (Kuznetsova *et al.*, 2002; Kalashnikova *et al.*, 2007; Zugle *et al.*, 2011). Therefore, synthesis and characterization of novel phthalocyanine complexes of lanthanides remain attractive to researchers, where diverse ratio of *Pc*/Ln could be expected. These lanthanide phthalocyanine derivatives have high intrinsic conductivity and interesting electrochemical behavior. Herein, we present the synthesis and structure of a phthalocyaninato erbium complex Er(C_32_H_16_N_8_)(C_12_H_8_N_2_)(C_5_H_7_O_2_). The complex is mirror-related and the central Er^III^ ion is located on the mirror plane. The Er^III^ ion is eight-coordinated to two O atoms from acetylacetone, two N (N_phen_) atoms from 1,10-phenanthroline and four isoindole N (N_iso_) atoms from the phthalocyanine ligand (Fig. 1). The Er—N distances are in the range of 2.376 (5)–2.529 (4) Å, and the Er—O distance is 2.272 (3) Å. The Er—N_iso_ bond distances are shorter than the Er—N_phen_ bond distances, but longer than the Er—O bond distances. The symmetry-related *Pc*^2-^ units are not parallel. The positive charge of the Er^III^ ion is balanced by the *Pc*^2-^ and acac^-^ groups. Strong π–π interactions (Fig. 2) could be found between the pyrrolyl group of *Pc* and the aromatic ring of 1,10-phenanthroline with a *Cg*1^ii^···*Cg*2 distance of 3.424 (3) Å and between the aromatic ring of the isoindole of *Pc* and the 1,10-phenanthroline with a *Cg*2···*Cg*3^ii^ distance of 3.657 (3) Å (*Cg*1, *Cg*2 and *Cg*3 are the centroids of the rings with atoms N5, C13, C14, C13^i^,C14^i^, atoms C17, C18, C22, C17^i^, C18^i^, C22^i^, and atoms C14–C16, C14^i^-C16^i^, respectively; symmetry codes: (i) *x*, -*y* + 3/2, *z*, (ii) 1 + *x*, *y*,*z*).

### Experimental

A mixture of Er(acac)_3_.H_2_O (0.0481 g, 0.10 mmol), dicyanobenzene (0.0512 g, 0.40 mmol), and DBU (0.076 g, 0.50 mmol) in *n*-pentanol (3 ml) was heated at 100 ° for 1.5 h under a slow stream of nitrogen (acac: acetylacetone; DBU: 1,8-diazabicyclo(5.4.0)undec-7-ene). After the volatiles were removed *in vacuo*, the residue was chromatographed on a silica gel column with CHCl_3_ with 2.5% of methanol (v:v) as the eluent to give a blue band containing Er(*Pc*)(acac)(phen) (23 mg, 20%). Single crystals suitable for X-ray analyses were obtained by slow diffusion of methanol into the chloroform solution of the complex. Calc. for C_49_H_31_ErN_10_O_2_: C 31.26, H 3.26, N 14.60. Found: C 31.20, H 3.24, N 14.61.

### Refinement

H atoms bound to C atoms were placed in calculated positions and treated as riding on their parent atoms, with C—H = 0.93 Å (aromatic C), C—H = 0.96 Å (methyl C) and with *U*_iso_(H) = 1.5U_eq_(C).

### Crystal data

**Table:**

[Er(C_32_H_16_N_8_)(C_5_H_7_O_2_)(C_12_H_8_N_2_)]	*F*(000) = 958
*M**_r_* = 959.10	*D*_x_ = 1.574 Mg m^−^^3^
Monoclinic, *P*2_1_/*m*	Mo *K*α radiation, λ = 0.71073 Å
Hall symbol: -P 2yb	Cell parameters from 15893 reflections
*a* = 9.913 (2) Å	θ = 6.2–54.9°
*b* = 16.887 (3) Å	µ = 2.13 mm^−^^1^
*c* = 12.622 (3) Å	*T* = 293 K
β = 106.72 (3)°	Block, purple
*V* = 2023.7 (7) Å^3^	0.28 × 0.22 × 0.17 mm
*Z* = 2	

### Data collection

**Table:**

Bruker SMART1000 CCD diffractometer	4769 independent reflections
Radiation source: fine-focus sealed tube	3876 reflections with *I* > 2σ(*I*)
Graphite monochromator	*R*_int_ = 0.050
Detector resolution: 0 pixels mm^-1^	θ_max_ = 27.5°, θ_min_ = 3.1°
ω scans	*h* = −12→12
Absorption correction: multi-scan (*SADABS*; Sheldrick, 2003)	*k* = −21→21
*T*_min_ = 0.576, *T*_max_ = 0.696	*l* = −16→16
19901 measured reflections	

### Refinement

**Table:**

Refinement on *F*^2^	Primary atom site location: structure-invariant direct methods
Least-squares matrix: full	Secondary atom site location: difference Fourier map
*R*[*F*^2^ > 2σ(*F*^2^)] = 0.033	Hydrogen site location: inferred from neighbouring sites
*wR*(*F*^2^) = 0.092	H-atom parameters constrained
*S* = 1.13	*w* = 1/[σ^2^(*F*_o_^2^) + (0.0219*P*)^2^ + 5.3744*P*] where *P* = (*F*_o_^2^ + 2*F*_c_^2^)/3
4769 reflections	(Δ/σ)_max_ < 0.001
287 parameters	Δρ_max_ = 2.01 e Å^−^^3^
0 restraints	Δρ_min_ = −2.24 e Å^−^^3^

### Special details

**Table:**

Geometry. All e.s.d.'s (except the e.s.d. in the dihedral angle between two l.s. planes) are estimated using the full covariance matrix. The cell e.s.d.'s are taken into account individually in the estimation of e.s.d.'s in distances, angles and torsion angles; correlations between e.s.d.'s in cell parameters are only used when they are defined by crystal symmetry. An approximate (isotropic) treatment of cell e.s.d.'s is used for estimating e.s.d.'s involving l.s. planes.
Refinement. Refinement of *F*^2^ against ALL reflections. The weighted *R*-factor *wR* and goodness of fit *S* are based on *F*^2^, conventional *R*-factors *R* are based on *F*, with *F* set to zero for negative *F*^2^. The threshold expression of *F*^2^ > σ(*F*^2^) is used only for calculating *R*-factors(gt) *etc*. and is not relevant to the choice of reflections for refinement. *R*-factors based on *F*^2^ are statistically about twice as large as those based on *F*, and *R*-factors based on ALL data will be even larger.

### Fractional atomic coordinates and isotropic or equivalent isotropic displacement parameters (Å^2^)

**Table:**

	*x*	*y*	*z*	*U*_iso_*/*U*_eq_	
Er1	0.56660 (3)	0.7500	0.21203 (3)	0.03343 (10)	
O1	0.5186 (3)	0.83217 (18)	0.0633 (3)	0.0439 (8)	
N1	0.6747 (6)	0.7500	0.4083 (5)	0.0414 (13)	
N2	0.7009 (4)	0.8908 (2)	0.4457 (3)	0.0474 (10)	
N3	0.4958 (4)	0.8662 (2)	0.2898 (3)	0.0380 (8)	
N4	0.2664 (4)	0.8910 (2)	0.1574 (3)	0.0406 (9)	
N5	0.3167 (5)	0.7500	0.1645 (5)	0.0375 (12)	
N6	0.7888 (4)	0.8299 (2)	0.2344 (3)	0.0425 (9)	
C1	1.0327 (7)	0.7909 (4)	0.7333 (6)	0.097 (3)	
H1A	1.0939	0.8181	0.7918	0.117*	
C2	0.9429 (6)	0.8332 (4)	0.6473 (6)	0.082 (2)	
H2A	0.9435	0.8883	0.6472	0.098*	
C3	0.8524 (5)	0.7913 (3)	0.5617 (4)	0.0537 (14)	
C4	0.7385 (5)	0.8153 (3)	0.4657 (4)	0.0432 (11)	
C5	0.5860 (5)	0.9128 (3)	0.3669 (4)	0.0421 (11)	
C6	0.5352 (5)	0.9944 (3)	0.3562 (4)	0.0479 (12)	
C7	0.5881 (7)	1.0633 (3)	0.4118 (5)	0.0688 (18)	
H7A	0.6729	1.0636	0.4678	0.083*	
C8	0.5102 (8)	1.1316 (3)	0.3812 (6)	0.086 (2)	
H8A	0.5440	1.1788	0.4169	0.103*	
C9	0.3831 (8)	1.1317 (3)	0.2985 (6)	0.0763 (19)	
H9A	0.3333	1.1788	0.2800	0.092*	
C10	0.3293 (6)	1.0629 (3)	0.2435 (5)	0.0584 (14)	
H10A	0.2431	1.0626	0.1890	0.070*	
C11	0.4085 (5)	0.9943 (3)	0.2722 (4)	0.0430 (11)	
C12	0.3848 (5)	0.9130 (2)	0.2331 (4)	0.0405 (11)	
C13	0.2344 (4)	0.8157 (3)	0.1314 (4)	0.0384 (10)	
C14	0.0918 (4)	0.7912 (3)	0.0671 (4)	0.0445 (11)	
C15	−0.0314 (5)	0.8337 (3)	0.0220 (4)	0.0534 (13)	
H15A	−0.0315	0.8888	0.0213	0.064*	
C16	−0.1538 (5)	0.7911 (3)	−0.0217 (5)	0.0614 (15)	
H16A	−0.2380	0.8181	−0.0517	0.074*	
C17	0.9133 (4)	0.7927 (3)	0.2813 (4)	0.0402 (10)	
C18	1.0409 (5)	0.8338 (3)	0.3219 (4)	0.0461 (12)	
C19	1.0361 (6)	0.9163 (3)	0.3109 (5)	0.0586 (15)	
H19A	1.1178	0.9460	0.3380	0.070*	
C20	0.9121 (5)	0.9531 (3)	0.2606 (5)	0.0600 (15)	
H20A	0.9089	1.0077	0.2508	0.072*	
C21	0.7899 (5)	0.9078 (3)	0.2238 (5)	0.0500 (13)	
H21A	0.7054	0.9336	0.1904	0.060*	
C22	1.1681 (5)	0.7899 (3)	0.3668 (4)	0.0546 (14)	
H22A	1.2522	0.8169	0.3966	0.066*	
C23	0.3996 (6)	0.8988 (3)	−0.1003 (5)	0.0666 (16)	
H23A	0.3257	0.9225	−0.0758	0.100*	
H23B	0.3651	0.8871	−0.1778	0.100*	
H23C	0.4774	0.9349	−0.0877	0.100*	
C24	0.4475 (5)	0.8234 (3)	−0.0367 (4)	0.0461 (12)	
C25	0.4112 (8)	0.7500	−0.0880 (6)	0.0535 (19)	
H25A	0.3588	0.7500	−0.1621	0.064*	

### Atomic displacement parameters (Å^2^)

**Table:**

	*U*^11^	*U*^22^	*U*^33^	*U*^12^	*U*^13^	*U*^23^
Er1	0.02919 (14)	0.02436 (13)	0.04208 (18)	0.000	0.00285 (11)	0.000
O1	0.0430 (17)	0.0388 (17)	0.045 (2)	−0.0011 (14)	0.0052 (15)	0.0044 (15)
N1	0.042 (3)	0.026 (2)	0.047 (3)	0.000	−0.002 (2)	0.000
N2	0.054 (2)	0.0318 (19)	0.047 (2)	0.0002 (18)	−0.0009 (19)	−0.0036 (17)
N3	0.0399 (19)	0.0301 (18)	0.041 (2)	−0.0003 (16)	0.0077 (16)	−0.0020 (16)
N4	0.0355 (19)	0.0347 (19)	0.050 (2)	0.0057 (16)	0.0099 (17)	0.0045 (17)
N5	0.027 (2)	0.034 (3)	0.050 (3)	0.000	0.009 (2)	0.000
N6	0.0366 (19)	0.0291 (18)	0.057 (3)	0.0018 (16)	0.0049 (17)	0.0027 (17)
C1	0.078 (4)	0.075 (4)	0.097 (5)	−0.003 (3)	−0.042 (4)	−0.008 (4)
C2	0.074 (4)	0.052 (3)	0.089 (5)	−0.002 (3)	−0.028 (4)	−0.013 (3)
C3	0.050 (3)	0.041 (3)	0.056 (3)	0.001 (2)	−0.009 (2)	−0.004 (2)
C4	0.042 (2)	0.034 (2)	0.047 (3)	0.002 (2)	0.003 (2)	−0.003 (2)
C5	0.049 (3)	0.030 (2)	0.043 (3)	0.000 (2)	0.006 (2)	−0.002 (2)
C6	0.063 (3)	0.029 (2)	0.047 (3)	0.005 (2)	0.007 (2)	0.000 (2)
C7	0.099 (5)	0.034 (3)	0.056 (4)	0.003 (3)	−0.005 (3)	−0.009 (2)
C8	0.131 (6)	0.031 (3)	0.075 (5)	0.009 (3)	−0.002 (4)	−0.011 (3)
C9	0.103 (5)	0.032 (3)	0.084 (5)	0.023 (3)	0.012 (4)	0.000 (3)
C10	0.072 (4)	0.035 (3)	0.064 (4)	0.013 (3)	0.013 (3)	0.006 (2)
C11	0.051 (3)	0.031 (2)	0.048 (3)	0.006 (2)	0.016 (2)	0.003 (2)
C12	0.040 (2)	0.027 (2)	0.054 (3)	0.0056 (19)	0.013 (2)	0.003 (2)
C13	0.032 (2)	0.037 (2)	0.045 (3)	0.0018 (18)	0.0104 (19)	0.003 (2)
C14	0.034 (2)	0.049 (3)	0.046 (3)	0.002 (2)	0.004 (2)	0.002 (2)
C15	0.040 (3)	0.060 (3)	0.054 (3)	0.010 (2)	0.004 (2)	0.006 (3)
C16	0.034 (2)	0.082 (4)	0.059 (3)	0.007 (2)	−0.001 (2)	0.001 (3)
C17	0.035 (2)	0.039 (2)	0.043 (3)	−0.0030 (19)	0.0060 (19)	−0.001 (2)
C18	0.035 (2)	0.052 (3)	0.049 (3)	−0.008 (2)	0.008 (2)	−0.003 (2)
C19	0.047 (3)	0.049 (3)	0.073 (4)	−0.019 (2)	0.007 (3)	−0.007 (3)
C20	0.053 (3)	0.034 (2)	0.088 (5)	−0.012 (2)	0.012 (3)	0.000 (3)
C21	0.041 (2)	0.035 (2)	0.071 (4)	0.000 (2)	0.010 (2)	0.003 (2)
C22	0.037 (2)	0.063 (3)	0.055 (3)	−0.010 (2)	−0.001 (2)	−0.003 (3)
C23	0.077 (4)	0.061 (4)	0.055 (4)	0.003 (3)	0.008 (3)	0.021 (3)
C24	0.037 (2)	0.048 (3)	0.050 (3)	0.001 (2)	0.008 (2)	0.007 (2)
C25	0.052 (4)	0.058 (5)	0.042 (4)	0.000	0.000 (3)	0.000

### Geometric parameters (Å, º)

**Table:**

Er1—O1^i^	2.272 (3)	C8—C9	1.386 (9)
Er1—O1	2.272 (3)	C8—H8A	0.9300
Er1—N5	2.376 (5)	C9—C10	1.379 (8)
Er1—N3^i^	2.388 (4)	C9—H9A	0.9300
Er1—N3	2.388 (4)	C10—C11	1.388 (6)
Er1—N1	2.400 (6)	C10—H10A	0.9300
Er1—N6	2.529 (4)	C11—C12	1.454 (6)
Er1—N6^i^	2.529 (4)	C13—C14	1.472 (6)
O1—C24	1.264 (6)	C14—C15	1.389 (6)
N1—C4	1.371 (5)	C14—C14^i^	1.393 (10)
N1—C4^i^	1.371 (5)	C15—C16	1.382 (7)
N2—C5	1.331 (6)	C15—H15A	0.9300
N2—C4	1.332 (6)	C16—C16^i^	1.388 (12)
N3—C5	1.366 (6)	C16—H16A	0.9300
N3—C12	1.377 (5)	C17—C18	1.404 (6)
N4—C13	1.330 (6)	C17—C17^i^	1.443 (9)
N4—C12	1.335 (6)	C18—C19	1.400 (7)
N5—C13^i^	1.369 (5)	C18—C22	1.432 (7)
N5—C13	1.369 (5)	C19—C20	1.361 (7)
N6—C21	1.323 (6)	C19—H19A	0.9300
N6—C17	1.359 (5)	C20—C21	1.394 (7)
C1—C1^i^	1.383 (13)	C20—H20A	0.9300
C1—C2	1.387 (8)	C21—H21A	0.9300
C1—H1A	0.9300	C22—C22^i^	1.346 (11)
C2—C3	1.384 (7)	C22—H22A	0.9300
C2—H2A	0.9300	C23—C24	1.508 (7)
C3—C3^i^	1.394 (10)	C23—H23A	0.9600
C3—C4	1.456 (6)	C23—H23B	0.9600
C5—C6	1.461 (6)	C23—H23C	0.9600
C6—C7	1.381 (7)	C24—C25	1.397 (6)
C6—C11	1.391 (7)	C25—C24^i^	1.397 (6)
C7—C8	1.380 (8)	C25—H25A	0.9300
C7—H7A	0.9300		
			
O1^i^—Er1—O1	75.28 (16)	C8—C7—C6	117.5 (5)
O1^i^—Er1—N5	80.57 (14)	C8—C7—H7A	121.3
O1—Er1—N5	80.57 (14)	C6—C7—H7A	121.3
O1^i^—Er1—N3^i^	79.71 (13)	C7—C8—C9	121.7 (5)
O1—Er1—N3^i^	145.58 (12)	C7—C8—H8A	119.1
N5—Er1—N3^i^	72.25 (11)	C9—C8—H8A	119.1
O1^i^—Er1—N3	145.58 (12)	C10—C9—C8	121.0 (5)
O1—Er1—N3	79.71 (12)	C10—C9—H9A	119.5
N5—Er1—N3	72.25 (11)	C8—C9—H9A	119.5
N3^i^—Er1—N3	110.52 (18)	C9—C10—C11	117.6 (5)
O1^i^—Er1—N1	141.10 (9)	C9—C10—H10A	121.2
O1—Er1—N1	141.10 (9)	C11—C10—H10A	121.2
N5—Er1—N1	112.6 (2)	C10—C11—C6	121.1 (5)
N3^i^—Er1—N1	70.88 (11)	C10—C11—C12	132.5 (5)
N3—Er1—N1	70.88 (11)	C6—C11—C12	106.4 (4)
O1^i^—Er1—N6	112.75 (13)	N4—C12—N3	128.2 (4)
O1—Er1—N6	74.62 (12)	N4—C12—C11	121.9 (4)
N5—Er1—N6	147.12 (8)	N3—C12—C11	109.8 (4)
N3^i^—Er1—N6	137.93 (12)	N4—C13—N5	128.4 (4)
N3—Er1—N6	82.25 (12)	N4—C13—C14	121.8 (4)
N1—Er1—N6	76.65 (15)	N5—C13—C14	109.6 (4)
O1^i^—Er1—N6^i^	74.62 (12)	C15—C14—C14^i^	121.1 (3)
O1—Er1—N6^i^	112.75 (13)	C15—C14—C13	132.3 (5)
N5—Er1—N6^i^	147.12 (8)	C14^i^—C14—C13	106.3 (3)
N3^i^—Er1—N6^i^	82.25 (12)	C16—C15—C14	117.6 (5)
N3—Er1—N6^i^	137.93 (12)	C16—C15—H15A	121.2
N1—Er1—N6^i^	76.65 (15)	C14—C15—H15A	121.2
N6—Er1—N6^i^	64.52 (16)	C15—C16—C16^i^	121.4 (3)
C24—O1—Er1	132.6 (3)	C15—C16—H16A	119.3
C4—N1—C4^i^	107.2 (5)	C16^i^—C16—H16A	119.3
C4—N1—Er1	123.2 (3)	N6—C17—C18	122.7 (4)
C4^i^—N1—Er1	123.2 (3)	N6—C17—C17^i^	117.5 (2)
C5—N2—C4	122.7 (4)	C18—C17—C17^i^	119.6 (3)
C5—N3—C12	107.5 (4)	C19—C18—C17	117.0 (4)
C5—N3—Er1	123.8 (3)	C19—C18—C22	123.8 (4)
C12—N3—Er1	123.0 (3)	C17—C18—C22	119.1 (5)
C13—N4—C12	122.8 (4)	C20—C19—C18	120.1 (5)
C13^i^—N5—C13	108.2 (5)	C20—C19—H19A	119.9
C13^i^—N5—Er1	124.3 (2)	C18—C19—H19A	119.9
C13—N5—Er1	124.3 (2)	C19—C20—C21	119.1 (5)
C21—N6—C17	118.1 (4)	C19—C20—H20A	120.4
C21—N6—Er1	123.5 (3)	C21—C20—H20A	120.4
C17—N6—Er1	117.0 (3)	N6—C21—C20	122.9 (5)
C1^i^—C1—C2	121.0 (4)	N6—C21—H21A	118.6
C1^i^—C1—H1A	119.5	C20—C21—H21A	118.6
C2—C1—H1A	119.5	C22^i^—C22—C18	121.2 (3)
C3—C2—C1	118.3 (6)	C22^i^—C22—H22A	119.4
C3—C2—H2A	120.9	C18—C22—H22A	119.4
C1—C2—H2A	120.9	C24—C23—H23A	109.5
C2—C3—C3^i^	120.8 (3)	C24—C23—H23B	109.5
C2—C3—C4	132.9 (5)	H23A—C23—H23B	109.5
C3^i^—C3—C4	106.2 (3)	C24—C23—H23C	109.5
N2—C4—N1	127.5 (4)	H23A—C23—H23C	109.5
N2—C4—C3	122.2 (4)	H23B—C23—H23C	109.5
N1—C4—C3	110.2 (4)	O1—C24—C25	124.2 (5)
N2—C5—N3	128.0 (4)	O1—C24—C23	115.6 (5)
N2—C5—C6	122.0 (4)	C25—C24—C23	120.2 (5)
N3—C5—C6	109.9 (4)	C24—C25—C24^i^	125.1 (7)
C7—C6—C11	121.1 (5)	C24—C25—H25A	117.5
C7—C6—C5	132.6 (5)	C24^i^—C25—H25A	117.5
C11—C6—C5	106.3 (4)		
			
O1^i^—Er1—O1—C24	24.4 (5)	C4^i^—N1—C4—C3	2.5 (8)
N5—Er1—O1—C24	−58.2 (4)	Er1—N1—C4—C3	−149.8 (4)
N3^i^—Er1—O1—C24	−20.3 (5)	C2—C3—C4—N2	−0.7 (11)
N3—Er1—O1—C24	−131.7 (4)	C3^i^—C3—C4—N2	174.2 (4)
N1—Er1—O1—C24	−172.7 (4)	C2—C3—C4—N1	−176.5 (7)
N6—Er1—O1—C24	143.6 (4)	C3^i^—C3—C4—N1	−1.6 (5)
N6^i^—Er1—O1—C24	90.4 (4)	C4—N2—C5—N3	−5.7 (9)
O1^i^—Er1—N1—C4	150.6 (3)	C4—N2—C5—C6	171.1 (5)
O1—Er1—N1—C4	−2.5 (6)	C12—N3—C5—N2	174.0 (5)
N5—Er1—N1—C4	−106.0 (4)	Er1—N3—C5—N2	−32.0 (7)
N3^i^—Er1—N1—C4	−166.4 (5)	C12—N3—C5—C6	−3.1 (6)
N3—Er1—N1—C4	−45.6 (4)	Er1—N3—C5—C6	150.8 (3)
N6—Er1—N1—C4	40.8 (4)	N2—C5—C6—C7	4.0 (10)
N6^i^—Er1—N1—C4	107.3 (5)	N3—C5—C6—C7	−178.7 (6)
O1^i^—Er1—N1—C4^i^	2.5 (6)	N2—C5—C6—C11	−175.2 (5)
O1—Er1—N1—C4^i^	−150.6 (3)	N3—C5—C6—C11	2.1 (6)
N5—Er1—N1—C4^i^	106.0 (4)	C11—C6—C7—C8	0.2 (10)
N3^i^—Er1—N1—C4^i^	45.6 (4)	C5—C6—C7—C8	−178.8 (7)
N3—Er1—N1—C4^i^	166.4 (5)	C6—C7—C8—C9	0.6 (11)
N6—Er1—N1—C4^i^	−107.3 (5)	C7—C8—C9—C10	0.0 (12)
N6^i^—Er1—N1—C4^i^	−40.8 (4)	C8—C9—C10—C11	−1.3 (10)
O1^i^—Er1—N3—C5	−153.5 (3)	C9—C10—C11—C6	2.1 (9)
O1—Er1—N3—C5	−109.7 (4)	C9—C10—C11—C12	179.5 (6)
N5—Er1—N3—C5	167.0 (4)	C7—C6—C11—C10	−1.6 (9)
N3^i^—Er1—N3—C5	104.5 (4)	C5—C6—C11—C10	177.7 (5)
N1—Er1—N3—C5	44.5 (4)	C7—C6—C11—C12	−179.6 (6)
N6—Er1—N3—C5	−34.0 (4)	C5—C6—C11—C12	−0.3 (6)
N6^i^—Er1—N3—C5	3.0 (5)	C13—N4—C12—N3	5.4 (8)
O1^i^—Er1—N3—C12	−3.4 (5)	C13—N4—C12—C11	−170.7 (5)
O1—Er1—N3—C12	40.4 (3)	C5—N3—C12—N4	−173.5 (5)
N5—Er1—N3—C12	−42.9 (3)	Er1—N3—C12—N4	32.2 (7)
N3^i^—Er1—N3—C12	−105.4 (3)	C5—N3—C12—C11	2.9 (5)
N1—Er1—N3—C12	−165.4 (4)	Er1—N3—C12—C11	−151.3 (3)
N6—Er1—N3—C12	116.1 (4)	C10—C11—C12—N4	−2.5 (9)
N6^i^—Er1—N3—C12	153.0 (3)	C6—C11—C12—N4	175.1 (5)
O1^i^—Er1—N5—C13^i^	40.5 (4)	C10—C11—C12—N3	−179.3 (5)
O1—Er1—N5—C13^i^	117.0 (5)	C6—C11—C12—N3	−1.6 (6)
N3^i^—Er1—N5—C13^i^	−41.6 (4)	C12—N4—C13—N5	−7.9 (8)
N3—Er1—N5—C13^i^	−160.9 (5)	C12—N4—C13—C14	166.2 (5)
N1—Er1—N5—C13^i^	−101.2 (4)	C13^i^—N5—C13—N4	171.1 (3)
N6—Er1—N5—C13^i^	158.2 (3)	Er1—N5—C13—N4	−28.3 (8)
N6^i^—Er1—N5—C13^i^	−0.7 (7)	C13^i^—N5—C13—C14	−3.5 (7)
O1^i^—Er1—N5—C13	−117.0 (5)	Er1—N5—C13—C14	157.1 (4)
O1—Er1—N5—C13	−40.5 (4)	N4—C13—C14—C15	−0.1 (9)
N3^i^—Er1—N5—C13	160.9 (5)	N5—C13—C14—C15	175.0 (6)
N3—Er1—N5—C13	41.6 (4)	N4—C13—C14—C14^i^	−172.9 (4)
N1—Er1—N5—C13	101.2 (4)	N5—C13—C14—C14^i^	2.2 (4)
N6—Er1—N5—C13	0.7 (7)	C14^i^—C14—C15—C16	0.6 (6)
N6^i^—Er1—N5—C13	−158.2 (3)	C13—C14—C15—C16	−171.4 (5)
O1^i^—Er1—N6—C21	114.3 (4)	C14—C15—C16—C16^i^	−0.6 (6)
O1—Er1—N6—C21	48.0 (4)	C21—N6—C17—C18	2.2 (8)
N5—Er1—N6—C21	5.6 (6)	Er1—N6—C17—C18	−164.8 (4)
N3^i^—Er1—N6—C21	−145.6 (4)	C21—N6—C17—C17^i^	−173.3 (4)
N3—Er1—N6—C21	−33.4 (4)	Er1—N6—C17—C17^i^	19.7 (4)
N1—Er1—N6—C21	−105.5 (4)	N6—C17—C18—C19	−0.9 (8)
N6^i^—Er1—N6—C21	173.1 (4)	C17^i^—C17—C18—C19	174.5 (4)
O1^i^—Er1—N6—C17	−79.5 (4)	N6—C17—C18—C22	−178.0 (5)
O1—Er1—N6—C17	−145.8 (4)	C17^i^—C17—C18—C22	−2.6 (6)
N5—Er1—N6—C17	171.8 (4)	C17—C18—C19—C20	−1.4 (9)
N3^i^—Er1—N6—C17	20.6 (4)	C22—C18—C19—C20	175.6 (6)
N3—Er1—N6—C17	132.8 (4)	C18—C19—C20—C21	2.2 (9)
N1—Er1—N6—C17	60.7 (3)	C17—N6—C21—C20	−1.3 (8)
N6^i^—Er1—N6—C17	−20.7 (4)	Er1—N6—C21—C20	164.8 (4)
C1^i^—C1—C2—C3	0.4 (9)	C19—C20—C21—N6	−0.9 (9)
C1—C2—C3—C3^i^	−0.4 (9)	C19—C18—C22—C22^i^	−174.3 (4)
C1—C2—C3—C4	173.9 (7)	C17—C18—C22—C22^i^	2.6 (6)
C5—N2—C4—N1	4.0 (9)	Er1—O1—C24—C25	−19.4 (8)
C5—N2—C4—C3	−171.0 (5)	Er1—O1—C24—C23	159.7 (4)
C4^i^—N1—C4—N2	−172.9 (3)	O1—C24—C25—C24^i^	0.2 (12)
Er1—N1—C4—N2	34.7 (8)	C23—C24—C25—C24^i^	−178.8 (5)

Symmetry code: (i) *x*, −*y*+3/2, *z*.

## References

[bb1] Bruker (2001). *SMART* Bruker AXS Inc., Madison, Wisconsin, USA

[bb2] Bruker (2003). *SAINT-Plus* Bruker AXS Inc., Madison, Wisconsin, USA

[bb3] Kalashnikova, I. P., Nefedov, S. E., Tomilova, L. G. & Zefirov, N. S. (2007). *Russ. Chem. Bull.* **56**, 2426–2432.

[bb4] Kuznetsova, N. A., Gretsova, N. S., Derkacheva, V. M., Mikhalenko, S. A., Solov’eva, L. I., Yuzhakova, O. A., Kaliya, O. L. & Luk’yanets, E. A. (2002). *Russ. J. Gen. Chem.* **72**, 300–306.

[bb5] Sheldrick, G. M. (2003). *SADABS* University of Göttingen, Germany.

[bb6] Sheldrick, G. M. (2008). *Acta Cryst.* A**64**, 112–122. 10.1107/S0108767307043930 18156677

[bb7] Zugle, R., Litwinski, C. & Nyokong, T. (2011). *Polyhedron*, **30**, 1612–1619.

